# Comparative analysis of SD biosensor standard^™^ M10 HPV and seegene anyplex^™^ II HPV HR for detecting high-risk human papillomavirus: a concordance study

**DOI:** 10.1186/s12879-025-10714-y

**Published:** 2025-03-03

**Authors:** Abigail Rembui Jerip, Vaenessa Noni, Vanessa Kiah Anthony, Andy Cheong Shin Bong, Jaria Adam, Cheng Siang Tan

**Affiliations:** 1 Department of Obstetrics and Gynaecology, Faculty of Medicine and Health Sciences, Universiti Malaysia Sarawak, Kota Samarahan, Sarawak 94300 Malaysia; 2 Department of Paraclinical Sciences, Faculty of Medicine and Health Sciences, Universiti Malaysia Sarawak, Kota Samarahan, Sarawak 94300 Malaysia; 3 Centre for Tropical and Emerging Diseases, Faculty of Medicine and Health Sciences, Universiti Malaysia Sarawak, Kota Samarahan, Sarawak 94300 Malaysia; 4 Pink and Teal Empowher, Kota Padawan, Kuching, Sarawak 93050 Malaysia

**Keywords:** Human papillomavirus, Cervical cancer, Point-of-care, Self-sampling, Standard M10 HPV, Anyplex

## Abstract

**Background:**

Cervical cancer, primarily caused by persistent high-risk human papillomavirus (hrHPV) infections, is a significant health burden, particularly in low-resource settings such as Sarawak, Malaysia. Effective prevention depends on effective vaccination and early hrHPV detection. This study compares the performance of the point-of-care test (POCT) SD Biosensor Standard™ M10 HPV and laboratory-based Seegene Anyplex™ II HPV HR assay, focusing on their ability to detect and genotype hrHPV in self-collected high vaginal swab samples.

**Methods:**

A total of 151 archived self-sampled high vaginal swabs from the Sarawak Urban and Rural Action for Cervical Cancer Elimination Programme (*Program SUARA*) were analyzed. hrHPV detection and genotyping were performed using Anyplex, which identifies 14 hrHPV genotypes, and M10, which detects HPV16, HPV18, and other hrHPV categorized into six genogroups. Agreement between the assays was evaluated using Cohen’s Kappa (κ), McNemar’s test, and overall agreement percentages. Statistical significance was determined with p-values, and discordant results were further analyzed for potential diagnostic implications.

**Results:**

The overall agreement between M10 and Anyplex for hrHPV detection was 92.05% (κ = 0.84, 95% CI 0.75–0.93), indicating almost perfect agreement. M10 demonstrated comparable sensitivity for detecting HPV16, HPV18, and other hrHPV genotypes, achieving 96.91% agreement (κ = 0.89, 95%CI 0.73-1.00) in hrHPV classification when discordant results were excluded. Genogrouping also showed almost perfect agreement (κ = 0.91, 95% CI 0.82–0.98). McNemar’s test indicated no significant difference in hrHPV detection rates (*p* > 0.05), affirming their comparable performance in detecting clinically significant hrHPV infections.

**Conclusion:**

The SD Biosensor Standard™ M10 HPV POCT and the Seegene Anyplex™ II HPV HR assay demonstrated almost perfect agreement in hrHPV detection and classification, supporting their complementary roles in cervical cancer prevention. M10’s rapid, field-deployable design makes it suitable for resource-limited settings, while Anyplex provides enhanced genotyping capability in laboratory environments, allowing informed vaccine strategy. Incorporating both assays into cervical cancer prevention programs can improve screening coverage and accessibility, particularly in underserved areas. These findings align with the World Health Organization’s cervical cancer elimination goals, reinforcing the importance of adaptable diagnostic tools in diverse healthcare contexts.

**Supplementary Information:**

The online version contains supplementary material available at 10.1186/s12879-025-10714-y.

## Background

Cervical cancer is predominantly driven by persistent infections with high-risk human papillomavirus (hrHPV) types, making it one of the most preventable cancers through comprehensive early screening and HPV vaccination. In Malaysia, cervical cancer ranks as the third most common cancer and is the second leading cause of cancer-related deaths among women [1]. In 2020, the age-standardized incidence rate of cervical cancer in Malaysia was 10.2 per 100,000 women, with Sarawak consistently recording the highest incidence at 12.1 per 100,000 [2]. These statistics underscore the urgency of improving vaccination coverage and screening, especially in regions with high incidence rates, as Malaysia works towards the World Health Organization’s (WHO) Cervical Cancer Elimination Initiative goal of reducing cervical cancer incidence to fewer than 4 cases per 100,000 women [3].

Sarawak, Malaysia’s largest region, faces significant healthcare access challenges, with more than half its population living in rural or hard-to-reach areas. These logistical barriers make reaching underserved populations essential for achieving cervical cancer elimination targets [2]. In response, Universiti Malaysia Sarawak (UNIMAS) in collaboration with Pink and Teal Empowher, a non-governmental organization and state government stakeholders and ministries, initiated HPV diagnostic efforts in 2018 with conventional Polymerase Chain Reaction (PCR) on clinician-collected cervical swabs [4]. In 2019, the programme expanded to include self-sampling and hrHPV detection using the careHPV system (Qiagen) [5], which was well-suited for low-resource settings [6]. However, the careHPV system lacked genotyping capability and internal control, limiting its effectiveness in providing a comprehensive profile of hrHPV genotype distribution in Sarawak. Furthermore, careHPV system was reported to be less sensitive when combined with self-sampling, especially among the older women [7].

To address these limitations, the careHPV system was replaced in 2023 with Seegene’s Anyplex™ II HPV HR system (Anyplex), offering both genotyping and an internal control. Yet, while Anyplex improved diagnostic depth, it relies on laboratory-based batch processing, reducing its applicability for point-of-care testing—a critical feature for effective screen-and-treat strategies in remote areas. In late 2024, the Standard M10 cartridge-based HPV test was adopted, providing a portable, sample-to-result solution in approximately one hour. This system not only detects HPV16, HPV18, and other hrHPV genotypes (grouped as G1-G6) but also enables field-based testing, directly supporting the *S*arawak *U*rban *a*nd *R*ural *A*ction for Cervical Cancer Elimination Programme (*Program SUARA*) [8], which aims to extend advanced hrHPV diagnostics to underserved communities.

To our knowledge, no comparative study has evaluated M10’s utility in detecting hrHPV. This study aims to evaluate the concordance between M10 HPV systems against Anyplex for hrHPV detection and genotyping in Sarawak. Through this comparison, we aim to identify optimal diagnostic strategies that enhance accessibility and accuracy in HPV testing, supporting Sarawak’s broader cervical cancer prevention initiatives.

## Materials and methods

### Specimens

The minimum sample size was estimated using Open Epi Sample size calculator (https://www.openepi.com/) at 139 samples, based on an expected agreement rate of 90%, a ± 5% margin of error, and a 95% confidence level. To ensure robustness, the study included 151 samples, which exceeded the calculated requirement.

The 151 archived self-sampled high vaginal swabs (HVSs) were obtained from *Program SUARA* HPV Laboratory at the Faculty of Medicine and Health Sciences, Universiti Malaysia Sarawak, Kota Samarahan, Sarawak, Malaysia. The mean age of the participants was 34.35 years (SD = 7.84, 24–64), with a median age of 33 years.

These samples consisted of 72 hrHPV positive and 79 hrHPV negative samples previously confirmed via Anyplex™ II HPV HR Detection (Seegene, South Korea) were included in this study Anyplex can simultaneously detects, differentiate, and quantifies 14 high-risk HPV genotypes (HPV16, 18, 31, 33, 35, 39, 45, 51, 52, 56, 58, 59, 66, 68) in a single reaction. The semiquantitative genotyping results were categorised into three levels corresponding to cyclic-Catcher Melting Temperature Analysis (CMTA) cycle 30 (+++), 40 (++), and 50 (+) [9]. The nucleic acid was extracted on a 48-well Genti™ Advanced Viral DNA/RNA Extraction kit (GenAll, South Korea), and detection was carried out on CFX96 DX Real-Time PCR Detection System (Bio-Rad, California, USA) (see Supplementary Material 1). These samples were anonymised and blinded to the operator. All tests were valid by the detection of the respective internal controls.

### POCT hrHPV

The samples were retested using the Standard M10 HPV system (SD Biosensor, South Korea) according to the manufacturer’s instruction (see Supplementary Material 1) for the qualitative detection of HPV16, 18 and other hrHPV (31, 33, 35, 39, 45, 51, 52, 56, 58, 59, 66, 68). The other 12 hrHPV were semi-genotyped into six [6] groups, namely G1 (HPV51-species α-5), G2 (HPV33, 52, 58-species α-9), G3 (HPV31, 35- species α-9), G4 (45, 59 species α-7), G5 (HPV39, 68 species α-7), and G6 (HPV56, 66- species α-6) [10]. This assay will be referred to as M10 throughout this manuscript.

### Data analyses

Firstly, we compared the performance of M10 [1] to detect hrHPV; [2] to differentiate hrHPV into HPV16, HPV18 and other hrHPV; and [3] genogrouping of other hrHPV to G1, G2, G3, G4, G5, G6 in comparison to the results obtained from Anyplex. All results were reclustered accordingly to allow for comparison. The flow of data and exclusion criteria is shown in Supplementary Material 2.

All results were tabulated and statistical analyses such as Agreement, Cohen’s Kappa (κ) and McNemar coefficients were carried out in SPSS Version 27. The interpretation of the Kappa (κ) coefficient was as follows: 0.81–1.00 indicated almost perfect agreement, 0.61–0.80 good agreement, 0.41–0.60 moderate agreement, 0.21–0.40 fair agreement, 0.00–0.20 minimal agreement, and less than 0.00 signified poor agreement between the assays [11].

## Results

### Detection of hrHPV

M10 and Anyplex have positive and negative agreement of 90.3% and 93.7%, respectively, which suggests that the results from both assays are in almost perfect agreement with one another (κ = 0.84, 95% CI: 0.75–0.93) and are largely consistent in their ability to detect hrHPV. McNemar’s test value (0.77; *p* > 0.05) indicates no significant difference between the assays. Both assays have an overall agreement of 92.05% (Table 1).

**Table 1:** Comparison of M10 (SD Biosensor) and anyplex (Seegene) for the detection of hrHPV

	Anyplex				
M10	NEG	POS	TOTAL	Cohen Kappa (κ) (*95% CI*)	McNemar
NEG	74	7	**81**	**0.84** (*0.75–0.93*)	**0.77** (*p > 0.05*)
POS	5	65	**70**		
**TOTAL**	**79**	**72**	**151**		

### Detection of HPV16, HPV18 and other hrHPV

We included 65 concordant samples in the calculation after excluding 7 discordant samples, the overall agreement between the assays was 96.91% (κ = 0.89, 95% CI: 0.73-1.00). McNemar’s Test’s (*p* > 0.05) shows no statistically significant difference in the discordance between the two tests. It shows that the performance of M10 and Anyplex was almost perfect in classifying hrHPV to HPV16, HPV18 and Others (Table 2).

**Table 2:** The comparison between M10 and anyplex in their ability to classify hrHPV into HPV16, 18 and Other

	Anyplex								
M10	16	18	16 and Other	16, 18	18 and Other	Other	TOTAL	Cohen’s kappa (κ) (95% CI)	McNemar
**16**	1						**1**	**0.89** *0.73-1.00*	**0.48** (*p > 0.05*)
**18**		5			1		**6**		
**16 & Other**			1				**1**		
**16**,** 18 & Other**				1			**1**		
**18 and Other**					1		**1**		
**Other**						55	**55**		
**TOTAL**	**1**	**5**	**1**	**1**	**2**	**55**	**65**		

### Non-HPV16/18 genogrouping

We subsequently removed 8 samples that were only positive for HPV16 and 18 and had incomparable discordant results. Of the 57 non-HPV16/18 samples by M10 and Anyplex, we found the overall genogrouping agreement to be 82.5% (κ = 0.91, 95% CI: 0.82–0.98) with almost perfect agreement in comparison with Anyplex. The McNemar’s Test (*p* > 0.05) suggests no significant difference between the two assays (Table 3).

**Table 3:** Comparison between M10 and anyplex in the classification of non-HPV16/18 hrHPV into G1, G2, G3, G4, G5 and G6

	Anyplex											
**M10**	**G1**	**G2**	**G3**	**G4**	**G5**	**G6**	**G1**,** G5**	**G2**,** G5**	**G4**,** G5**	**TOTAL**	**Cohen Kappa (**κ) *95% CI*	**McNemar**
**G1**	9									**9**	**0.91** *0.82–0.98*	**1.0**(*p > 0.05*)
**G2**		18								**18**		
**G3**			1							**1**		
**G4**				4						**4**		
**G5**					8					**8**		
**G6**						7				**7**		
**G1**,** G2**		2								**2**		
**G1**,** G5**					1		3			**4**		
**G2**,** G4**		1								**1**		
**G2**,** G5**								2		**2**		
**G4**,** G5**									1	**1**		
**TOTAL**	**9**	**21**	**1**	**4**	**9**	**7**	**3**	**2**	**1**	**57**		

### Performance

The 7 discordant samples performed on M10 were monoinfections with CTMA + [(HPV16 (x2), 52 (x2), 58, 39, 51)]. The discordance may be due to the lower sensitivity of M10 compared to Anyplex. It must be noted that 2 monoinfection samples contain HPV16. However, M10 detected 5 more positives that were not detected by Anyplex. These samples can be grouped into G2 (60%), G5 (20%) and G6 (20%) with the mean Ct value of 20.66 (95% CI, *p* < 0.05, Standard Deviation (SD) of 11.72, 9–33) is not close to the manufacturer-set negative cutoff value of Ct > 39. It is worth noting that two G2 samples as determined by M10 had the Ct < 9 and were missed by Anyplex. The actual HPV genotypes missed by Anyplex cannot be elucidated without further unbiased sequence analyses.

Discordances in classifying hrHPV into HPV16, HPV18, and other hrHPV have no clinical impact per our screen-and-treat strategy, as all hrHPV-positive women will be triaged for cervical examination. However, such distinctions may still be informative.

## Discussion

Our study aimed to compare the performance of the point-of-care test Standard™ M10 HPV System (SD Biosensor, South Korea) (referred to as M10) against the Anyplex™ II HPV HR Detection (Seegene, South Korea) (referred to as Anyplex). Both assays are CE-IVD certified, and compliant with Malaysia’s Medical Device Act (MDA). Since 2023, the *SUARA Program*’s laboratory at Universiti Malaysia Sarawak has adopted the Anyplex assay in combination with self-sampling, especially within remote rural settlements, employing a screen-and-treat strategy where feasible.

This screen-and-treat approach begins with community outreach for self-collected samples, which are then tested in batches using the Anyplex assay as a triaging tool. Individuals testing positive for high-risk HPV (hrHPV) are recalled for a secondary triage-and-treat stage, which includes colposcopy and thermocoagulation as necessary [12]. In contrast, for walk-in patients, a point-of-care test (POCT)-and-treat approach is more practical. M10 can address diagnostic gaps on-site, eliminating the need for repeating the two-stage process, and in return ensuring timely results and treatment.

Our findings revealed nearly perfect agreement between M10 and Anyplex, with an overall agreement of 92.05% (κ = 0.84). This performance is comparable to the Xpert HPV (Cepheid), another cartridge-based POCT, which demonstrated an agreement of 91.5% (κ = 0.844) against Anyplex28, a broader Seegene assay designed to detect additional low-risk HPV types [13]. When Anyplex28 and Xpert HPV were evaluated against the GP5+/6 + EIA comparator HPV assay, sensitivity and specificity were 98.3% and 93.6% for Anyplex28, and 94.1%, and 90.3% for Xpert HPV, respectively [14, 15]. These data suggest that cartridge-based POCTs may exhibit slightly lower agreement compared to laboratory-based assays [15].

Each HPV DNA assay, however, has unique sensitivity and specificity profiles. For example, the Anyplex assay has been reported with sensitivities ranging from 92.5 to 98.3% and specificities from 81.7 to 94.1% for detecting cervical intraepithelial neoplasia grade 2 or worse (CIN2+) [16, 17, 18]. These performance metrics highlight the balance between sensitivity and specificity in correlating HPV positivity to cytological pathology [19]. While M10 demonstrated slightly lower positive agreement for hrHPV detection than Anyplex, its clinical utility as a triaging tool in resource-limited areas remains significant. By providing accessible and timely screening options, M10 has the potential to expand coverage and save lives. Notably, the careHPV system—though falling short of international standards when used with self-sampling [15] has been successfully implemented in low-resource settings in Cambodia and Tanzania [7, 20]. It is essential to recognize that HPV DNA-based testing represents a paradigm shift in cervical cancer screening, moving from pathology-based detection to identifying the etiological agent. Numerous studies affirm that HPV DNA testing is more sensitive than cytology in detecting precancerous lesions and cervical cancers [21, 22, 23] and any HPV DNA test is better than none.

The WHO’s Cervical Cancer Elimination Initiative outlined a three-pronged strategy to eliminate cervical cancer as a public health threat: vaccination, screening, and treatment [3, 24]. The first prong aims to vaccinate 90% of girls under 15 with HPV vaccines. The second prong emphasises screening 70% of women for hrHPV by the age of 35 and again by 45 using validated HPV DNA tests. The third prong ensures that 90% of women diagnosed with precancer or cancer receive appropriate treatment. The use of POCT may ensure that hrHPV-positive women receive appropriate care and treatment within the same day. To address variability in sensitivity and specificity across assays, women testing negative for hrHPV may benefit from retesting within 3–5 years using a validated assay from the same or different manufacturer.

### Limitations

Despite having a significant sample size, our samples can be expanded in a population study to improve the power. Our posthoc selection of positive and negative samples may introduce bias but allows a higher number of positive samples to be evaluated for the concordance study. This study focused solely on the concordance between two assays without correlating hrHPV detection with cytological or histopathological outcomes. Anyplex is a clinically validated assay that conforms to international standards for primary cervical cancer screening, the diagnostic implications of discordant cases remain uncertain without follow-up cytology or colposcopy. While cotesting offers no additional benefit over stand-alone hrHPV DNA testing, it may result in higher colposcopy referrals due to false positives [23]. In our study, all volunteers positive for hrHPV by M10 but previously negative by Anyplex were recalled for cervical examinations. Future concordance studies integrating clinical follow-up would better assess the impact of using M10 in real-world screening program.

## Conclusion

The Standard M10 (SD Biosensor, South Korea) and Anyplex II HPV HR (Seegene, South Korea) were found to be in almost perfect agreement to detect hrHPV in high vaginal swabs. No one assay can be regarded as superior or inferior as both have demonstrated their strength and weaknesses.

## Electronic supplementary material

Below is the link to the electronic supplementary material.

Supplementary Material 1

## Data Availability

All data generated or analyzed during this study are included in this article.

## Abbreviations

CE-IVD: European Conformity-in vitro diagnostic
CIN: Cervical intraepithelial neoplasia
Cl: Confidence level
Ct: Cycle threshold
CMTA: Cyclic-Catcher Melting Temperature Analysis
DNA: Deoxyribonucleic acid
HPV: Human papillomavirus
hr: High-risk
MDA: Medical Device Act
PCR: Polymerase chain reaction
POCT: Point-of-care-test
RNA: Ribonucleic acid
SUARA: Sarawak Urban and Rural Action for the Elimination of Cervical Cancer
UNIMAS: Universiti Malaysia Sarawak
WHO: World Health Organisation
κ: Kappa
