# Supplementary material for: Comparative analysis of SD biosensor standard™ M10 HPV and seegene anyplex™ II HPV HR for detecting high-risk human papillomavirus: a concordance study
Source: BMC Infect Dis. 2025 Mar 3;25:304. doi: 10.1186/s12879-025-10714-y (PMC11877861; doi:10.1186/s12879-025-10714-y)
Supplement: Supplementary file 1 — Supplementary Material 1 [file 12879_2025_10714_MOESM1_ESM.docx]

# Supplementary material 1: Detailed protocols for Anyplex and M10 according to the manufacturers’ instructions.

1. ***Specimen collection***

A total 151 archived self-sampled high vaginal swabs (HVSs) were collected using the FLOQSwab (Copan, Italy), with patients given instructions on the self-sampling technique by trained staff, according to the instructions given by the manufacturer. Exclusion criteria include: i) unmarried women; ii) older than 65 years old and above; iii) pregnant with known pregnancy and iv) hysterectomised women. Dry self-sampled HVSs were then transported at room temperature to the laboratory, and stored in 4℃ until further use.

1. ***hrHPV detection using the Anyplex™ II HPV HR Detection***

Prior to HPV testing, dry self-sampled HVSs were resuspended in 3mL Phosphate-buffered saline (PBS) (FirstBase, Malaysia) and vortexed for 30s. Nucleic acid was extracted from 200µL of resuspension using the 96-well Genti™ Advanced Viral DNA/RNA Extraction kit (GenAll, South Korea) and carried out on the GENTI™ ^32^ Advanced Automatic Extraction Equipment (GenAll, South Korea).

Subsequently, HPV detection was performed using the Anyplex™ HR Detection (Seegene, South Korea) assay and carried out on the CFX96 DX Real-time thermocycler (Bio-Rad, USA) with each PCR performed in 20µL of reaction mix and 5µL of extracted nucleic acid. The thermal conditions consisted of initial incubation of 50℃ for 4 mins, denaturation at 95℃ for 15 mins, 50 cycles of denaturation at 95℃ for 30s, annealing at 60℃ for 1 min and elongation at 72℃ for 30s.

The assay uses the human beta-globin gene for as its internal control while co-amplifying the targeted L1 gene of the 14 hrHPV types simultaneously (Oštrbenk et al., 2018). All remaining cell suspension were then stored in -20℃ until further use.

1. ***POCT hrHPV detection using the SD Biosensor M10***

The remaining suspension was then retested using the Standard M10 HPV system (SD Biosensor, South Korea) according to the manufacturer’s instructions. Briefly, 1.4mL of cell suspension was pre-treated using the STI sample pre-treatment kit (SD Biosensor, South Korea) and placed into the Standard M10 HPV cartridge (SD Biosensor, South Korea) where real-time PCR amplification assay was performed in the STANDARD M10 module (SD Biosensor, South Korea). The assay simultaneously amplifies the 14 hrHPV E6/E7 HPV oncogene for detection, with the internal control assessment done to test the presence of the human hydroxymethylbilane synthase (HMBS) endogenous gene and to ensure that the reagents in the cartridge are functioning properly.

**Reference**

Oštrbenk, A., Xu, L., Arbyn, M. and Poljak, M. (2018). Clinical and analytical evaluation of the Anyplex II HPV assay with the VALGENT-3 framework. Journal of Clinical Microbiology, 56(11), 1-18. 10.1128/JCM.01176-18

# Supplementary Material 2: Sample flow and exclusion criteria

**57 hrHPV**

A total of 57 hrHPV datasets from Table 2 were used in the comparative analysis to classify non-HPV16 and 18 to their respective genogroups (G1-G6).

Eight datasets were excluded because they contained only HPV16 and/or 18 or lacked non-HPV16/18.

(Table 3)

**65 hrHPV**

A total of 65 hrHPV datasets from Table 1 were used in the comparative analysis to classify hrHPV to HPV16, 18 and other-hrHPV

(Table 2)

**65 Pos by M10**

65 concordant samples were included in the comparative analyses for hrHPV detection.

(Table 1)

**151 samples**

72 Pos, 79 Neg by Anyplex

**END**

**81 Neg by M10**

7 discordant datasets

**70 Pos by M10**

5 discordant datasets were excluded due to incomparable Anyplex results
